# Machine learning and comparative genomics approaches for the discovery of xylose transporters in yeast

**DOI:** 10.1186/s13068-022-02153-7

**Published:** 2022-05-20

**Authors:** Mateus Bernabe Fiamenghi, João Gabriel Ribeiro Bueno, Antônio Pedro Camargo, Guilherme Borelli, Marcelo Falsarella Carazzolle, Gonçalo Amarante Guimarães Pereira, Leandro Vieira dos Santos, Juliana José

**Affiliations:** 1 Genomics and Bioenergy Laboratory (LGE), Institute of Biology, University of Campinas (UNICAMP), Campinas, São Paulo 13083-970 Brazil; 2 Genetics and Molecular Biology Graduate Program, Institute of Biology, University of Campinas (UNICAMP), Campinas, Brazil; 3 Microforge Ltd., Av Prefeito José Lozano Araújo 1136, Paulínia, São Paulo 13140-558 Brazil; 4 Senai Innovation Institute for Biotechnology, São Paulo, 01130-000 Brazil

**Keywords:** Xylose, Xylose transporter, Machine learning, Feature selection, Pentose metabolism, Industrial biotechnology

## Abstract

**Background:**

The need to mitigate and substitute the use of fossil fuels as the main energy matrix has led to the study and development of biofuels as an alternative. Second-generation (2G) ethanol arises as one biofuel with great potential, due to not only maintaining food security, but also as a product from economically interesting crops such as energy-cane. One of the main challenges of 2G ethanol is the inefficient uptake of pentose sugars by industrial yeast *Saccharomyces cerevisiae*, the main organism used for ethanol production. Understanding the main drivers for xylose assimilation and identify novel and efficient transporters is a key step to make the 2G process economically viable.

**Results:**

By implementing a strategy of searching for present motifs that may be responsible for xylose transport and past adaptations of sugar transporters in xylose fermenting species, we obtained a classifying model which was successfully used to select four different candidate transporters for evaluation in the *S. cerevisiae hxt-null* strain, EBY.VW4000, harbouring the xylose consumption pathway. Yeast cells expressing the transporters SpX, SpH and SpG showed a superior uptake performance in xylose compared to traditional literature control Gxf1.

**Conclusions:**

Modelling xylose transport with the small data available for yeast and bacteria proved a challenge that was overcome through different statistical strategies. Through this strategy, we present four novel xylose transporters which expands the repertoire of candidates targeting yeast genetic engineering for industrial fermentation. The repeated use of the model for characterizing new transporters will be useful both into finding the best candidates for industrial utilization and to increase the model’s predictive capabilities.

**Graphical Abstract:**

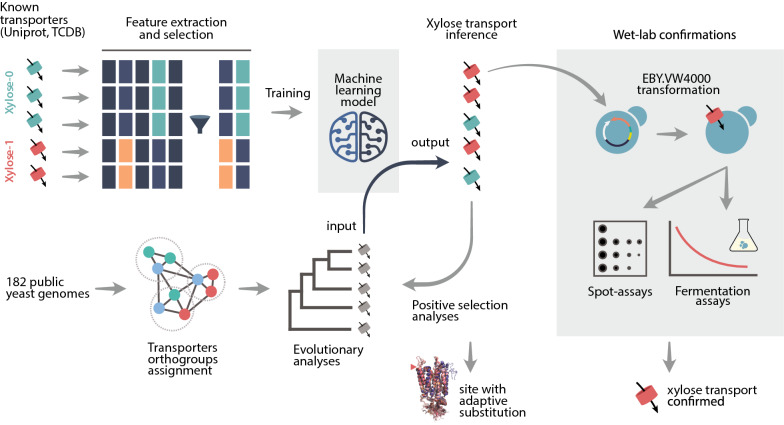

**Supplementary Information:**

The online version contains supplementary material available at 10.1186/s13068-022-02153-7.

## Background

For the last few decades, the scientific community has expended efforts to find cleaner energy alternatives to the fossil-based matrix as a means to mitigate the consequences of climate change from the use of said fossil fuels. One strategy is the use of biofuels produced from lignocellulosic biomass, also called second-generation (2G) biofuels. This strategy is desirable, as lignocellulose is found in the cell walls of all plants and allows many different matrices to be used industrially [1–4].

Plant cell walls comprise mainly cellulose (30–50%), hemicellulose (25–3%) and lignin (15–20%) [5]. The 2G process involves breaking down these main saccharide fractions into their monomers, predominantly glucose and xylose, that can then be metabolized by microorganisms into different bioproducts, and in the context of biofuels, bioethanol [6–8]. 2G biofuels appear as a promising driver on energy security due to it not competing directly with the food industry and not needing more plantations to achieve energy security [8].

Xylose consumption follows two pathways: the first, deemed the oxidoreductive pathway, comprises a conversion of xylose into xylitol through the enzyme xylose reductase, followed by a conversion of xylitol into xylulose by xylitol dehydrogenase. The second, called the isomerase pathway, comprises a one-step conversion of xylose into xylulose by xylose isomerase. Both pathways then have a conversion of xylulose into xylulose-5-phosphate by xylulokinase which then follows the pentose phosphate pathway [9].

The main organism used industrially for these biotechnological applications is the yeast *Saccharomyces cerevisiae* due to its resistance to inhibitors, high product yield, and ease of manipulation [9, 10]. However, its utilization of xylose naturally is lacking, requiring genetic engineering steps to insert one of the two xylose metabolism pathways to ethanol. Although yeast strains with these pathways are already used extensively, the challenge of xylose consumption remains, related to the cofactor imbalance on the oxidoreductive pathway, the need to further engineer or evolve exogenous xylose isomerases on the isomerase pathway, or inhibition of the xylose pathway by glucose due to sugar phosphorylation mechanisms [9, 11–14].

Regarding xylose transport, *S. cerevisiae* has many hexose transporters that are also capable of transporting xylose, such as the Hxt family of transporters and Gal2 [15–17]. Many xylose transporters have been found in other yeast species and considered candidates for industrial use by engineering *S. cerevisiae*, such as Sut1-3 and Xut1 from *Scheffersomyces stipitis* [18], Gxs1 and Gxf1 from Candida intermedia [19] and XylHP from *Debaryomyces hansenii* [20]. Details of their kinetic properties in xylose and glucose have also been described [15, 21–24]. Besides yeasts, one of the most studied and known xylose transporters is xylE from *Escherichia coli* [25–27].

Xylose consumption rates decrease when coupled with glucose due to competition of these sugars by endogenous transporters for access to the transport system, where first the organism depletes all the hexoses in its media, and only then slowly metabolizes xylose [15, 28, 29]. Even though many xylose-transporting proteins have been described in literature, this inefficient consumption pattern remains and what defines the ability to transport xylose is not entirely understood. Also, sequence, evolutionary or chemical interaction characteristics (hereafter discussed as features) behind transport capacity are still not completely understood [27], as much variability on transport capacity, velocity and affinity is seen, one example is the sugar transporter Gxf1, which shows an efficiency shift at certain sugar concentrations [23].

Many studies have been done to describe new transporters from new species [19, 20, 30–33], engineer hexose or pentose transporters for better efficiency through genetic engineering and directed evolution [28, 34–40], develop transporter testing yeast strains [16, 30, 41], resolving crystallographic structures coupled with xylose [25], but the main genomic drivers for xylose affinity, such as adaptive evolutionary signals (e.g. positive selection and convergent evolution), structural affinity and relations between the key residues already described as important for transport have not been found. This is in part due to xylose transport not having a single structural motif indicating its trait and no known specific transporters, even though many amino acid sites for different transporters have been described to be key for xylose affinity [15, 40, 42]. Also, as transporters have evolved in a multi-genic strategy (gene duplication, resulting in multiple sequences coding for the same protein) as an evolutionary solution to increase throughput and adaptation [43], this makes it harder to choose, test and find the best candidates for industrial purposes. Understanding these kinetic dynamics is also desirable for better rational engineering of yeasts. One novel promising approach has been to understand the evolutionary history of xylose consuming yeasts compared to non-consumers, finding genomic adaptations that may have arisen in response to the need of using xylose [44, 45]. A similar genome-wide comparative genomics study searching for adaptations in key xylose utilization pathway was previously described [46]. A similar approach using comparative genomics focused on the phylogenetic structure was used to prospect and choose novel xylose transporter candidates from *Candida sojae* [47].

The use of machine learning models to classify and predict has been previously applied to transporters as a means to separate and differentiate functional classes and families [48, 49], however due in part to the many classes in which transporters fall into, the models often lack accuracy and precision. Simpler methods, such as sequence homology, topological comparison or sequence profiling have been used before to describe different proteins, including sugar transporters [48, 50–54], but a unified process that weights each methods’ importance has not been described. The goal of this study was to cross sequence pattern information by extracting different features from known annotated xylose transporters in yeast or bacteria with past evolutionary adaptations via comparative genomics of 182 yeast genomes as an attempt to describe what genomic elements define if a sugar transporter is capable to transport xylose or not. A classification model was created and successfully used against sugar transporter families from the dataset to find potential xylose transporters. These candidates were then characterized by growing yeast expressing these candidate genes on a set of different sugars. Finally, the structure of each of these four transporters was modelled and their docking pose coupled with glucose and xylose was compared against the crystallographic structure of the known symporter from *E. coli* xylE.

In this work, we believe another step was given on facilitating the search for xylose transporters and understanding what the main drivers for xylose affinity are, while presenting a model that can already help to choose the most likely xylose-transporting candidates to take on for wet-lab work, and that with further use will become even more reliable.

## Results

### Sugar transporters from 182 yeasts cluster in 4 families

We selected 182 genomes (Additional file 1: Table S1) from the *Saccharomycotina* clade available for download in NCBI to try and understand the evolutionary history and adaptations of different yeasts that conferred an ability or not to ferment or consume xylose (manuscript in preparation). Orthofinder [55] analysis followed by recovery of families of interest through BLAST with known xylose transporters as baits revealed that sugar transporters grouped into 4 orthogroups: families 9, 10, 1180 and 7608, containing 1298, 1293, 204 and 8 genes, respectively. Full protein sequences for each family are available as Additional file 4: File S1.

### Training and testing dataset

The dataset for model selection comprised sugar transporters for fungi and bacteria as annotated and registered in Uniprot [56] and on TCDB [57]. Xylose transporters were carefully screened from these data, and due to insufficient proteins with this function, a literature search was done to increase their number. In total, 396 proteins, from which 25 were able to transport xylose, had their amino acid sequence retrieved and were used for machine learning (Additional file 2: Table S2 is given with the gene name, Uniprot ID and publication describing xylose transport). The data were split into training and test sets using scikit-learn’s [58] *train_test_split*.

**Fig. 1:**
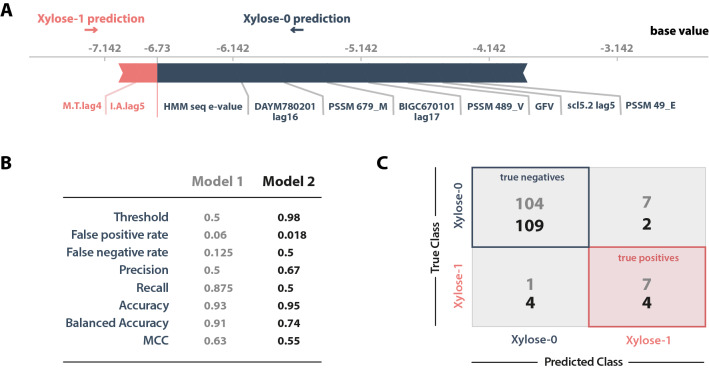
Graphical representations of machine learning model against the dataset. **a** Force-plot of most important features as calculated by Recursive Feature Elimination by Cross-Validation with XGBoost. Features highlighted in red are responsible for driving the final prediction of a sample into the positive category (A probable xylose transporter) while features in blue drive the prediction into the negative category (A non-xylose transporter). The base value represents the average prediction for the samples, while the size of the feature represents its impact (higher or lower importance). **b** Common metrics used to evaluate a model, the grey values correspond to the base threshold model and blue to the altered threshold. **c** Confusion matrix showing the results of predictions against the test data

From the more than 30,000 features extracted for the sequences, 13 were defined by the model as most important, from which 2 were impactful for a xylose transport capacity classification (Xylose-1), and the other 11 for an inability to transport xylose (Xylose-0) (Fig. 1a). Most of these features are derived from profile-based descriptors, these include the Position Scoring Matrices Features (PSSM), which indicate patterns of different sequences and scores each amino acid according to its position on the sequence, and are useful for predicting function of sites or classifying residues [59], the two features that drive the prediction to xylose-1 and the custom Hidden Markov Model (HMM), which similarly to PSSM calculates and scores sequence position, sites and patterns given other known or similar sequences, that was extracted from the non-cytoplasmic domains of the sequences, the latter also being the feature with the most impact. Other important features are related to relative mutability (DAYM780201) [60], residue volume and its consequence for the final protein conformation (BIGC670101) [61], modelling possible ligand–target interaction (scl5.2lag.5) [62] and adding more information, such as hydrophobicity in relation to near residues, to the amino acid composition (Pc1.c) [63].

Due to the dataset imbalance (only 25 out of 396 were xylose-transporting proteins), statistical oversampling techniques were implemented to reduce this imbalance. Standard classification metrics were done to evaluate the model, such as receiver operating characteristic (ROC) and precision-recall graphs, in addition to the confusion matrix which allow visualization of the absolute number of samples in each class correctly or incorrectly predicted by the model. The ROC curve showed higher increments of true positive rate than of the false positive rate, which means that the model efficiently classified the positive samples from the testing dataset (AUC = 0.95 for both classes) without losing much precision. Similarly, the precision-recall analysis showed an average precision of 0.73. However, due to the initial imbalance against the positive class, and to reduce overfitting issues arising from oversampling, we sought to remove this bias by analysing the data more attentively, modifying how results were judged and giving more weight to precision. As the default model (Model 1) classification threshold is 0.5 to assign a sample to each class (0–0.49 as negative; 0.5–1 as positive), we manually edited (Model 2) so that only samples with prediction probabilities of 0.98 or higher were classified as xylose transporters. At the cost of classification power for true xylose transporters (lower recall), we were able to almost nullify false positives for this class and thus increase precision and decrease the false positive rate (Fig. 1b).

### Choosing transporter candidates from the comparative genomics dataset

Four transporter families were returned during our phylogenomics analysis by searching the 182 yeasts dataset families against know sugar transporters with xylose-transporting capacity (XUT1, GXF1, GXS1, HXT7, Xylhp, XUT3, xylE, Cs3894, Cs4130) through BLAST and the MFS HMM from PFAM database [64]. All sequences from these families underwent feature extraction as done for the training and testing dataset and were tested against the model with altered baseline threshold. 25 sequences were predicted as xylose transporters, from which four sequences were chosen to be tested experimentally: Spaxylofer2423 (SpX), Spagorwiae6242 (SpG), Spahagerda5424 (SpH) and Suglignoha2156 (SuL), from *Spathaspora xylofermentans*, *Spathaspora gorwiae*, *Spathaspora hagerdaliae and Sugiyamaella lignohabitans* species, respectively. These sequences were chosen as three of them are from the known xylose fermenting *Spathaspora* genus, and the *Sugiyamaella lignohabitans* species, which is also known to consume xylose while not being part of the fermenter’s clade [46]. SuL was identified as the *HXT5*/*HTX6* hexose transporter from *Sugiyamaella lignohabitans* [65], SpX, SpH and SpG as *HXT2* from *Spathaspora sp.* [66] or *HXT5* from *Candida subhashii* [67], through BLAST search.

All 25 sequences were from fam10. Interestingly, known xylose transporters such as Gxf1 (Caninterme1096), Cs4130 (Cansojae5099) and Cs3894 (Cansojae522) from *Candida intermedia* and *Candida sojae*, respectively, were also part of fam10, which highlights the potential of homologs in other yeast species that are seldom explored. Caution was taken when the model had not displayed these known xylose transporters in its output, however, on closer inspection of the prediction probabilities, this happened due to the increased restriction on the classification (a 0.95–0.96 threshold would have included them).

**Fig. 2:**
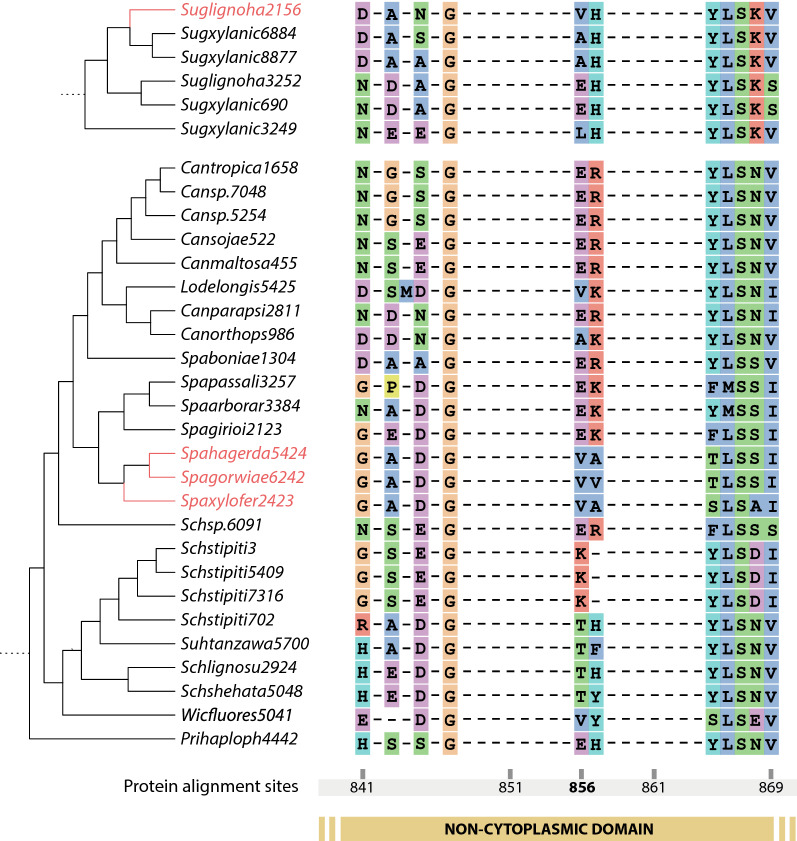
Snippet of fam10 phylogeny transformed into a cladogram for visualization purposes, coupled with the alignment around the site found under positive selection by MEME. In red are the transporters chosen for further characterization. Bootstraps are not shown as all of them on these clades were over 80

Additionally, five of these 25 sequences were found to have positive selection evidence on one codon, at protein alignment site 856, which by Interpro analysis and posteriorly by 3D modelling, was observed to be positioned on the extracellular non-cytoplasmic domain of the first helix. This result indicates that these proteins had amino acid substitutions potentially functioning as adaptations related to the xylose fermenting phenotype through their recent evolution and thus emphasizes the importance of these sequences on xylose metabolism. Due to the size of fam10 and the heterogeneity of transporters contained in it, the alignment on this site was mostly indels for most species, however, many interesting patterns were found at the positively selected codon. Firstly, being part of the non-cytoplasmic region, this site was contemplated on the HMM feature, which was also the most impactful for the machine learning model. Secondly, as can be seen in Fig. 2, the four previously chosen candidates for experimental validation have the same amino acid (valine) at the positively selected site. While this might be expected for the three *Spathaspora* candidates and explained by it probably being an adaptation inherited from their common ancestor during speciation, the *Sugiyamaella* transporter also contains valine at this site while also having diverged from the clade containing *Spathaspora* much earlier during these yeasts’ evolutionary history. This pattern might indicate convergent evolution at this site.

### Evaluation of chosen transporters in different sugars

The substrate uptake capacity from these four sugar transporters was evaluated in the strain EBY_Xyl1, a modified yeast strain derived from EBY.VW4000 [16] lacking most of its hexose sugar transporters, rendering it unable to grow on most sugars except maltose, and engineered with the xylose oxidoreductive pathway genes. The four transporters were codon-optimized for expression in *S. cerevisiae* (Additional file 5: File S2), assembled with the promoter and terminator sequences of the *TDH1* gene from the glycolytic pathway and cloned into the multi-copy vector pRS426. The xylose-facilitator *GXF1* from *C. intermedia* was cloned in the same manner as the four candidates and used as a positive control for xylose transport, since this transporter is one of the best heterologous xylose transporters described in literature [23, 68].

**Fig. 3:**
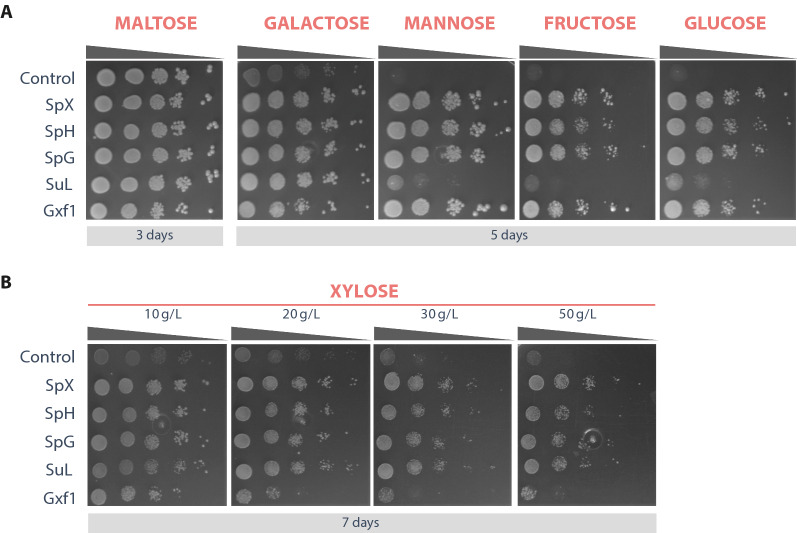
Spot-assay of EBY_Xyl1 carrying each of the indicated transporters and growing in **a** different sugars and **b** different concentrations of xylose. Initial OD_600_ was settled at 1 before the tenfold serial dilution. Plates were incubated in 30 °C. All experiments were performed in triplicate

We analysed the substrate range of EBY_Xyl1 mutants carrying the specified sugar transporters using six different sugars on solid culture medium—2 % maltose (control), mannose, fructose, glucose, and galactose. The transformants were grown for 24 h to the exponential phase on Maltose and spotted in tenfold serial dilutions onto solid culture medium. All transporters, except for SuL, were able to confer growth of EBY_Xyl1 on all sugars, indicating a substrate promiscuity commonly seen for sugar transporters. However, SuL was especially surprising as growth in fructose, mannose and glucose was almost non-existent (Fig. 3a).

The growth of EBY_Xyl1 mutants carrying transporter genes and Gxf1 as positive control were also compared in solid medium with 1, 2, 3 and 5% of xylose as the sole carbon source. All transporters were able to confer growth in all sugar concentrations, and the four transporters showed higher growth than Gxf1 at higher xylose concentrations (Fig. 3b).

### Yeast fermentation with chosen xylose transporters

**Fig. 4:**
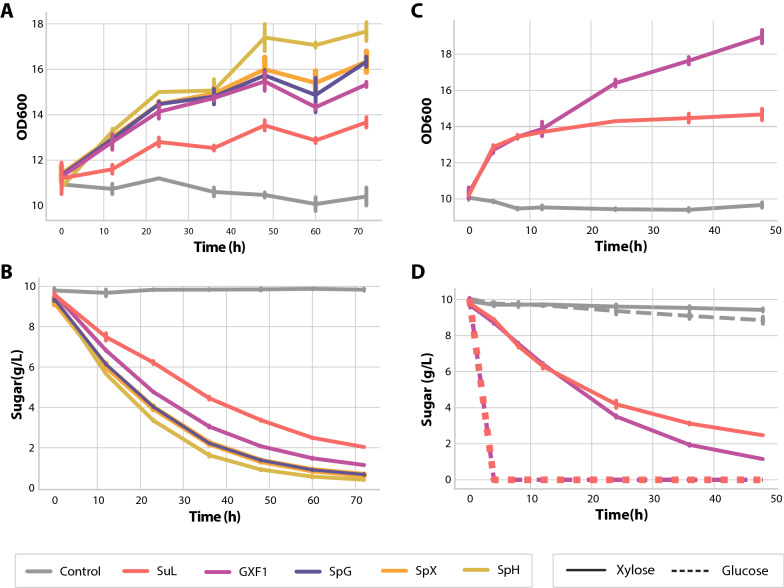
Comparative fermentation assays of EBY_Xyl1 expressing different transporters in xylose (full lines) or glucose (dashed lines). **a** Growth of EBY_Xyl1 during xylose fermentation. **b** Xylose consumption of EBY_Xyl1 cells expressing the transporters over time. Note that SpX does not appear clearly as it overlaps with SpG. **c** Growth of EBY_Xyl1 expressing SuL, *GXF1* as positive control and pRS426 (empty vector) as negative control during xylose/glucose co-fermentation and **d** sugar consumption of EBY_Xyl1 expressing SuL, *GXF1* as positive control and pRS426 (empty vector) as negative control during xylose/glucose co-fermentation (note that SuL glucose fermentation overlaps with *GXF1*)

Fermentation assays were done in EBY_Xyl1 for the four transporters in media containing 1% xylose as the sole carbon source, as well as for Gxf1 and empty pRS426 vector (positive and negative controls, respectively). Based on the results shown in Fig. 4, SpX, SpG and SpH conferred superior growth capability compared to the traditional Gxf1 transporter. Cells expressing SuL had a smaller rate of growth. A similar pattern was seen for xylose consumption, where SpH conferred a slightly higher assimilation rate than the other transporters, and SuL being the slowest.

Due to the performance results from the spot-assay in different C6 sugars, simultaneous consumption of xylose and glucose by SuL was evaluated by fermentation of a mixture of 10 g/L each of xylose and glucose., Glucose was entirely consumed on the first 4 h of experiment, while xylose was slowly consumed during the same period, only increasing after glucose depletion. After 20 h, cells expressing GXF1 also demonstrated more efficiency in transporting xylose than SuL.

### Comparative docking of transporters

**Fig. 5:**
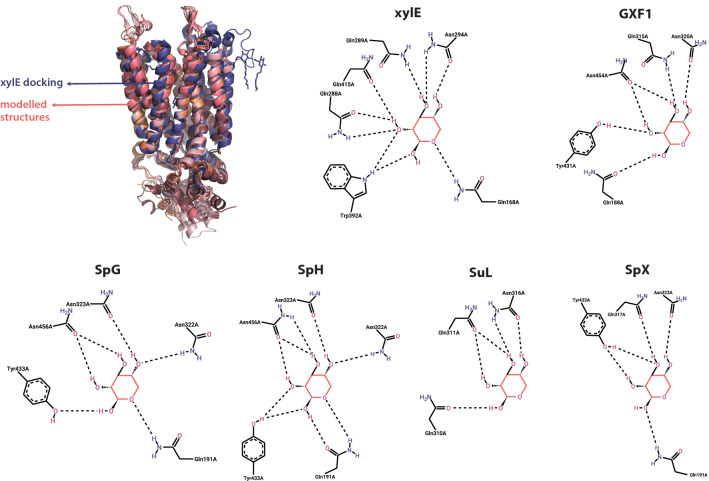
Superimposed structures of xylE coupled with xylose (blue) and predicted structures for the four xylose transporters and GXF1 (pink tones). The 2D representations show the probable interactions between xylose and amino acids in the binding site for each transporter

All four transporters and Gxf1 were modelled through RoseTTAFold in Robetta server [69] for comparative docking using the *xylE* crystallographic structure bound to xylose or glucose as a comparison basis [25]. Figure 5 and Table 1 outline the docking results when compared to the pose of the ligands on the crystal (lowest root-mean-square deviation of atomic positions—RMSD, the average distance between superimposed structures—obtained between the docked pose and the crystal’s ligand pose during self-docking) and their simulated pose. Near identical poses for all transporters were achieved, with RMSDs ranging from 0.6 to 2 Å, which are generally accepted as good modelling outcomes [70]. All sequences had similar affinity to xylose, with SuL having the lowest, SpX, SpH and SpG slightly higher than Gxf1, and *xylE* having the highest. These results are partially supported by the comparative fermentation in xylose, in which these affinity patterns can be seen on xylose consumption rate and cell growth. All transporters’ calculated docking affinity to glucose was higher than to xylose, indicating the typical behaviour of substrate promiscuity and preferential uptake of glucose.

**Table 1:** Docking results for the four candidate transporters, xylE (self-docking) and Gxf1

Protein name	Xylose		Glucose	
	Affinity	Δ RMSD from crystal	Affinity	Δ RMSD from crystal
xylE	− 5.8	1.776	− 6	0.619
SuL	− 5.0	0.948	− 5.8	1.166
Gxf1	− 5.3	1.085	− 5.7	1.296
SpH	− 5.4	2.274	− 5.5	2.252
SpX	− 5.5	2.454	− 6	2.668
SpG	− 5.5	2.300	− 5.6	1.223

Affinity represents the stability of the ligand in the binding site (the more negative the better), and ΔRMSD represents the difference in pose between docked prediction and xylE crystal position

## Discussion

Describing novel transporters is an important step to help unravel the underlying causes in which a sugar transporter is able to transport xylose while another does not show this capacity. The use of computational approaches, such as with machine learning or comparative genomics, have become powerful tools in this search effort. Some algorithms have been proposed to predict different transporter classes based on their function to facilitate classification and categorization [54, 71], however, even though they efficiently categorize membrane transporters, these models aim for a broader classification, which results in not deep enough information regarding function for some specific purposes such as sugar transport capacity discrimination. This work presents a classification model with the purpose of distinguishing sugar transporters in their ability to transport xylose.

The use of oversampling techniques coupled with increasing the prediction threshold were able to create a trustful model which identified 25 potential xylose transporters, from which four were experimentally validated. This shows that, even with a restrictive baseline threshold, many transporters from a diverse group of species were returned, highlighting the potential of different microorganisms, many of them rarely studied with an applied biotechnological view, in supplying candidate genes for bio-industrial applications. Regarding other sequences not chosen for further investigation, some transporters were surprising to appear as positive from our model, such as Pickudriav5544 and Pickudriav5977 from *Pichia kudriavzevii*, which on a first literature screen for the 182 yeast phylogenetics study appears as a species incapable of xylose transport. A second screening showed that they are able to utilize xylose [72], which increased confidence that the altering of the threshold for the models’ predictions effectively removed false positives.

The model also highlighted 13 features as most important for its predictive capability, from which two, PSSM profiling and AAindex, were also used found in previous studies [71]. Interestingly, the model also highlighted the HMM score feature, originally developed in this work. Briefly, this feature was generated by isolating the non-cytoplasmic region of the known xylose transporters used for model creation through sequence alignment followed by comparison with the InterproScan results for *Debaryomyces fabryi* Xylhp (Uniprot accession Q64L87). Another interesting feature was GFV tripeptides, which are located on transmembrane portions of the transporters, but their direct relation to xylose transport is unclear. Nonetheless, all predicted transporters had this tripeptide conformation ranging from 1 to 3 groups. PSSM and the custom HMM features highlight and hint that there is a hidden motif associated with xylose affinity, which due to the nature of the boosting algorithm was not yet humanly interpretable, but with future improvements of the model may come to light. These in silico results are also in accordance with previous experimental works that have also shown that xylose affinity is correlated with sequence alterations, key motifs, and amino acid interactions with the sugar ligand [15, 37].

One interesting pattern that we detected posteriorly to choosing the candidates was that SpX, SpG and SpH were on the same clade on the fam10 phylogeny and also form a monophyletic clade on the species’ phylogeny, highlighting an overlap of past adaptations (phylogenomic analysis) and recent patterns (machine learning analysis). This pattern overlap can also be seen on the probable convergent evolution of the site found under positive selection between the 3 *Spathaspora* chosen candidates and the *Sugiyamaella* transporter, as *Sugiyamaella* diverged much earlier but still has the same adaptations as the *Spathaspora* transporters. Moreover, the HMM feature was created on the non-cytoplasmic domains of the known xylose transporters and the site under positive selection is also on one of these non-cytoplasmic domains, again highlighting an overlap between evolutionary marks and more recent sequence attributes. As mentioned before, this site is located on the N-terminal region of the first transmembrane helix, which may have some function associated with stabilizing the rocker-switch mechanism when the transporter is active. Mutating this residue in future studies could help to understand more its role for sugar transport, as structural studies of MFS transporters, such as xylE, have focused on mutating amino acids associated with the predicted sugar binding sites [25, 73, 74]. While machine learning and comparative genomics have been used before separately to classify or describe transporters, to our knowledge this is the first study that associates both strategies and apply them to a bio-industrial challenge.

Regarding the experimental validation, spot-assay results were surprising for SuL, as there was almost no growth in C6 sugars glucose, fructose, and mannose, while growth in xylose and galactose was restored. Yeasts expressing the four candidates also showed greater growth compared to Gxf1 on concentrations above 10 g/L of xylose indicating that the chosen candidates could be viable for industrial use, as lignocellulosic biomass contains higher xylose concentrations than the condition where Gxf1 is comparable to the other proteins [75, 76], which translates into a higher xylose concentration during industrial fermentations [77] where these transporters have greater activity. However, future studies using *S. cerevisiae* strains adapted to industrial conditions would be required to further validate these candidates. Fermentation assays were also interesting, as SpX, SpG and SpH were all slightly more efficient than the widely used Gxf1 transporter, with SuL lagging. Co-fermentation assays of SuL and Gxf1 showed that the latter has a slightly superior consumption pattern of xylose, using glucose during the first 4 h, and only then using xylose, as expected. Gxf1 also conferred higher growth in the C5 sugar. Again however, an evaluation on an industrial strain with higher xylose and glucose concentrations on the media would reveal the industrial potential of these candidates, as coupled with spot-assay results these patterns suggest that these four xylose transporters can expand the repertoire for industrial use and build a strong case for the model’s use on prospecting novel candidates. Also, even though SuL showed a lower consumption rate both in xylose and during co-fermentation when compared to Gxf1, the lack of growth in glucose and fructose as seen in the spot-assay is a rare phenotype for sugar transporters and could indicate an interesting target for mutagenesis or directed evolution aiming to increase its xylose consumption rate. By using a combination of sugar transporters with different affinities, a future industrial yeast strain could metabolize C6 and C5 sugars more effectively, increasing the viability of the 2G process.

As an attempt to consolidate all results obtained in this study, the 3D structure of the experimentally evaluated transporters was created, with docking analysis coupled with glucose and xylose. Docking analysis successfully modelled both xylose and glucose poses for the evaluated transporters, which gave us a bigger confidence on the affinity calculations. These affinity results were confirmed during fermentation, where slight growth differences were seen in accordance with the slight differences in predicted affinity.

Finally, with future advances on describing novel xylose-transporting proteins and the increase of sequenced genomes, the model can be improved and become an important tool for researchers on helping to prospect industrial candidate transporters.

## Conclusions

2G ethanol is a promising energy matrix alternative for current and future needs. One challenge for this technology’s viability is an efficient and uninhibited transport of pentose sugars into yeast cells, which drives the search for novel and capable xylose transporters to be engineered into industrial *S. cerevisiae*, the main organism used for this kind of fermentation. The coupled machine learning and comparative genomics approach presented here yielded several xylose transporter candidates, from which four were experimentally tested against a wide range of sugars. The dimensionality reduction by feature selection highlighted that the most important features were related to HMM and PSSM profiles, indicating that xylose transport can in part be explained by amino acid patterns in the non-cytoplasmic domains of the proteins, especially the pore and binding sites, a result also seen in previous point mutation studies and descriptions of known transporters’ structures, indicating the conformity of the model with previous studies in literature. All transporters tested successfully transported xylose, most of them in rates superior to the traditional Gxf1, one of the best-known heterologous xylose transporters in literature, and all conferred higher cell growth at larger xylose concentrations. Docking analysis showed a similar pattern, with SuL having the lowest affinity to xylose and the other three transporters having a slightly higher affinity than Gxf1.

For future studies, the model’s predictive capability should be provided with data arising from new xylose transporter characterizations, as well as attempts to create models by adding information of known xylose transporters from other organisms, such as *Arabidopsis thaliana*. We believe that not only researchers interested in prospecting novel xylose-transporting candidates for industrial application can already make use of the model to aid their selection of best targets for wet-lab evaluation, but also understanding xylose transport on a broader scale then fungi and bacteria, with the help of this model, it will be possible to better understand and reveal the intricacies of xylose transport.

## Methods

### 182 *genomes dataset*

Genomes were retrieved from NCBI, based on if they were the representative genome for that species. 30 genomes had no coding sequences prediction, and so had their genes predicted by using AUGUSTUS 3.3.2 [78] and GeneMark-ES Suite 4.32 [79] separately and reconciled with Evidence Modeler 1.1.1 [72]. Genes were also filtered by their longest ORF via Transdecoder and by having at least 80 amino acids in the sequence. Genome completeness and success of gene prediction was analysed by utilizing the BUSCO v3 [80] Saccharomycotina dataset, which comprised conserved genes from this group and must be found on the data for a successful gene prediction. Additional file 6: fig. S1 shows the results for the BUSCO analysis.

### Phylogenomic analysis

Genes were clustered into gene families by means of Orthofinder 2.2 [55]. Transporter families were retrieved through the HMM MFS_1 and MFS_5 profiles from Pfam [64], and through known xylose transporters (XUT1, GXF1, GXS1, HXT7, Xylhp, XUT3, xylE, Cs3894, Cs4130) as baits for a BLAST search. Multi-sequence alignments were undertaken with MAFFT [81] for protein sequences (L-INS-i), and with MACSE 2.01 [82] by anchoring with the protein alignment for CDS. Alignments were trimmed using Trimal 1.4.1 [83] for phylogenetic inferences of the conserved domains, as due to the nature of the dataset (too many sequences from heterogenous groups) there were many gaps. Phylogenetic inferences were done through Maximum Likelihood with IQTree 1.6.12 [84] running 1000 bootstraps. Selection analysis was done by marking sequences that were output from the Machine Learning model as foreground and running HYPHY MEME 2.0.1 [85].

### Machine learning

Machine learning modelling usually undergoes the following steps: data clean-up and division into training and testing datasets, feature extraction and selection, model training, and evaluation. After clean-up, all intermediate steps are done on the training dataset and evaluation is done with the testing dataset. This separation of training and testing allows for a faithful evaluation of a model’s metrics by isolating some of the data in such a way that testing is done on part of the dataset upon which the model has no bias. Also, all these steps can be done using different machine learning algorithms and it is recommended to test several models using different algorithmical approaches and selecting the best performant. Feature extraction in the case of protein modelling represents decomposing the amino acid sequence into different descriptors that either mathematically explain the sequence or highlight some trait of interest, while feature selection is used for dimensionality reduction, computational optimization and highlighting the importance of certain features for classification.

Sugar transporters from fungi and bacteria were retrieved from Uniprot and TCDB databases. CD-HIT [86] was done to remove proteins with more than 80% sequence similarity, except for the known xylose transporters (experimentally validated by other studies), which were manually re-added to the dataset if removed. Xylose transporter sequences with their respective publication are shown in Additional file 2: Table S2. Most features were extracted with the protr package [87], which generates many numerical explainers of a given protein sequence. Also, an HMM feature was calculated by aligning the xylose transporters and using the sequence of Xylhp from *Debaryomyces fabryi* (Uniprot accession Q64L87) to predict domains and important sites through Interproscan; then, the non-cytoplasmic domains and probable sugar binding sites were isolated from the alignment and the HMM profile was created. We assumed as all sequences are aligned, the binding sites would be roughly in the same position. Other features added were the PS0021 and PS00217 sugar transport signatures from PROSITE database [88] using ScanProsite [89], the protein existence evidence, which sugars it transports, protein annotation, and if there is evidence in literature for xylose transport.

Following feature extraction and clean-up, sequences were divided into training and testing datasets through scikit-learn’s 0.21.2 [58] *train_test_split* with 70% used for training and 30% for testing.

Feature selection was done by Recursive Feature Elimination with Cross-Validation (RFECV) by using a Gradient Boosting Decision Tree classifier, implemented by XGBoost 0.82 [90]. Feature importance visualization was done using Yellowbrick 0.9.1 [91] or SHAP 0.29.3 explainers [92].

Some statistical transformations using oversampling were attempted to mitigate dataset imbalance, at the cost of some overfitting of the data. Additional file 7: figure S2 shows UMAP 0.3.9 [93] spatial distribution of samples after oversampling through Random Oversampling, SMOTE, SMOTEEEN, SMOTETomek and ADASYN. Except for Random Oversampling, all these transformers use a nearest neighbour approach to add a synthetic new sample to the data, which is related to the parameters of its neighbors. A model from all these attempts was made, however only SMOTEEEN was taken further as the evaluated model metrics were more satisfactory.

Model evaluation was done through usual metrics, such as accuracy, precision-recall, AUC, ROC curve, Balanced Accuracy and MCC, however, we were also attentive to brute numbers, because of the dataset imbalance distorting metric results. False positives were penalized by increasing the classification threshold of the xylose-positive class to 0.98, and this restrictive model was used for choosing candidates. The four transporter families from our phylogenomics dataset had the 13 most important features calculated and the model was ran. Sequences were chosen based on our knowledge if their respective species is a known fermenter or consumer of xylose.

All code used for model creation and data engineering can be found at https://gitlab.com/Matt_BF/Xylose_Transporter_ML.

### Strains and constructions

Strain EBY_Xyl1 was constructed from EBY.VW4000 by inserting an expression cassette containing the genes *XYL1* and *XYL2* from *S. stipitis* and an additional copy of xylulokinase (*XKS1*) under control of different promoters of the glycolytic pathway of *S. cerevisiae* as previously described [47]. Synthesized SpG, SpH, SpX and SuL were cloned into pRS426 at the EcoRI and NotI sites flanked with the promoter and terminator regions from *THD1* gene and further transformed into EBY_Xyl1 through the LiAc/SS-DNA/PEG protocol [94]. Transformants were selected in YNB medium lacking uracil. The transformation was confirmed by PCR using primers for the coding sequence of each gene (Additional file 3: Table S3).

### Media and culture conditions

Yeast cells were grown on liquid YP medium (10 g/L yeast extract and 20 g/L peptone) supplemented with 20 g/L D-glucose (YPD) for cell propagation or 20 g/L D-xylose (YPX) for xylose growth analysis. Transformed cells were grown at 30 °C in complete synthetic media YNB (6.7 g/L yeast nitrogen base without amino acids, Difco) supplemented with 1 g/L drop-out without uracil, 20 g/L glucose and 20 g/L agar [75]. YP was autoclaved at 121 °C for 20 min and YNB was filter-sterilized using 0.2-μm bottle-top filters. Strain EBY.VW4000, kindly supplied by Prof. Eckhard Boles from Goethe university [39], and strain EBY_Xyl1 were grown in YNB with D-maltose instead of D-glucose.

### Fermentations

Yeast strains were pre-grown on YNB supplemented with 5 g/L of casamino acids (Difco), 1 g/L of tryptophan (Sigma) and 50 g/L of d-maltose for 24 h. Cells were then harvested by centrifugation, washed three times with sterile water and resuspended to an OD_600_ of 10. Fermentation experiments were performed aerobically in 250 mL Erlenmeyer flasks using 70 mL of YNB supplemented with 5 g/L of casamino acids, 1 g/L of tryptophan (Sigma) and 10 g/L of xylose. For simultaneous glucose and xylose co- fermentation, 10 g/L of both sugars were used. The cells were incubated at 30 °C/200 rpm. Experiments were performed in triplicate and samples were collected to measure optical density and for HPLC analysis.

### Molecular docking

Molecular docking analysis was done using Autodock-Vina 1.1.2, ran via UCSF Chimera 1.15. Transporter structures for Gxf1, SuL, SpG, SpH and SpX were modelled through ROSETTAFold via the Robetta server [69], with the lowest angstrom error estimate models chosen for docking, and the glucose and xylose ligands were obtained from PubChem (IDs 5793 and 135191, respectively). The xylE crystal structure bounded to xylose (PDB code 4GBY) or glucose (PDB code 4GBZ) was used as the reference for self-docking and for interpretation of the other transporters (evaluation and comparison of ligand position on the candidate transporters and during self-docking, as in the closest the ligand poses during docking to the pose from the xylE crystal the better). Ten docking runs were done for each transporter and the one with the lowest RMSD from the xylE crystal was chosen. Comparison of ligand position and poses was done with DockRMSD [95]. Visualization of docking results and ligand positions was done with pyMOL, and the 2D ligand interactions were extracted on the Protein-Plus web server [96].

## Supplementary Information

**Additional file 1:** **Table S1.** Information of the species used for comparative genomics, including xylose fermentation or consumption capacity, and associated publication describing.

**Additional file 2: Table S2.** Xylose transporters used as positive samples for machine learning, including Uniprot accession, organism and publication describing xylose utilization.

**Additional file 3: Table S3.** Primers used to validate transformation of EBY_Xyl1.

**Additional file 4: File S1.** *S. cerevisiae* Codon optimized sequences for the four transporters chosen for characterization in xylose.

**Additional file 5: File S2.** FASTA files of the four transporter families from the comparative genomics analysis.

**Additional file 6: Figure S1.** BUSCO results for gene prediction.

**Additional file 7: Figure S2.** UMAP spatial distribution of data after oversampling.

## Data Availability

All data generated or analysed during this study are included in this published article and its Additional information files.

## References

[1] Zaldivar J, Nielsen J, Olsson L (2001). Fuel ethanol production from lignocellulose: a challenge for metabolic engineering and process integration. Appl Microbiol Biotechnol.

[2] Gírio FM, Fonseca C, Carvalheiro F, Duarte LC, Marques S, Bogel-Łukasik R (2010). Hemicelluloses for fuel ethanol: a review. Biores Technol.

[3] Dias MOS, Junqueira TL, Cavalett O, Pavanello LG, Cunha MP, Jesus CDF (2013). Biorefineries for the production of first and second generation ethanol and electricity from sugarcane. App Energy.

[4] Balat M (2011). Production of bioethanol from lignocellulosic materials via the biochemical pathway : a review. Energy Convers Manag.

[5] Zhao Z, Xian M, Liu M, Zhao G (2020). Biochemical routes for uptake and conversion of xylose by microorganisms. Biotechnol Biofuels.

[6] Maga D, Thonemann N, Hiebel M, Sebastião D, Lopes TF, Fonseca C (2019). Comparative life cycle assessment of first- and second-generation ethanol from sugarcane in Brazil. Int J Life Cycle Assess.

[7] Gírio FMM, Fonseca C, Carvalheiro F, Duarte LCC, Marques S, Bogel-Łukasik R (2010). 2010 Hemicelluloses for fuel ethanol: a review. Biores Technol.

[8] dos Santos LV, de Barros Grassi MC, Gallardo JCM, Pirolla RAS, Calderón LL, de Carvalho-Netto OV (2016). Second-generation ethanol: the need is becoming a reality. Ind Biotechnol.

[9] Jeffries TW (2006). Engineering yeasts for xylose metabolism. Curr Opin Biotechnol.

[10] Botstein D, Fink GR (2011). Yeast: an experimental organism for 21st century biology. Genetics.

[11] Cunha JT, Soares PO, Romaní A, Thevelein JM, Domingues L (2019). 2019 Xylose fermentation efficiency of industrial *Saccharomyces cerevisiae* yeast with separate or combined xylose reductase/xylitol dehydrogenase and xylose isomerase pathways. Biotechnol Biofuels.

[12] Hua Y, Wang J, Zhu Y, Zhang B, Kong X, Li W (2019). Release of glucose repression on xylose utilization in Kluyveromyces marxianus to enhance glucose-xylose co-utilization and xylitol production from corncob hydrolysate. Microb Cell Fact.

[13] Lane S, Xu H, Oh EJ, Kim H, Lesmana A, Jeong D (2018). Glucose repression can be alleviated by reducing glucose phosphorylation rate in *Saccharomyces cerevisiae*. Sci Rep.

[14] Brink DP, Borgström C, Persson VC, Osiro KO, Gorwa-Grauslund MF (2021). D-xylose sensing in *Saccharomyces cerevisiae*: Insights from D-glucose signaling and native D-xylose utilizers [Internet]. Int J Mol Sci.

[15] Farwick A, Bruder S, Schadeweg V, Oreb M, Boles E (2014). Engineering of yeast hexose transporters to transport D-xylose without inhibition by D-glucose. Proc Natl Acad Sci.

[16] Wieczorke R, Krampe S, Weierstall T, Freidel K, Hollenberg CP, Boles E (1999). Concurrent knock-out of at least 20 transporter genes is required to block uptake of hexoses in *Saccharomyces cerevisiae*. FEBS Lett.

[17] Hamacher T, Becker J, Gárdonyi M, Hahn-Hägerdal B, Boles E (2002). Characterization of the xylose-transporting properties of yeast hexose transporters and their influence on xylose utilization. Microbiology.

[18] Weierstall T, Hollenberg CP, Boles E (1999). Cloning and characterization of three genes (SUT1–3) encoding glucose transporters of the yeast Pichia stipitis. Mol Microbiol.

[19] Leandro MJ, Gonçalves P, Spencer-Martins I (2006). Two glucose/xylose transporter genes from the yeast Candida intermedia : first molecular characterization of a yeast xylose–H + symporter. Biochem J.

[20] Ferreira D, Nobre A, Silva ML, Faria-Oliveira F, Tulha J, Ferreira C (2013). XYLH encodes a xylose/H+ symporter from the highly related yeast species Debaryomyces fabryi and Debaryomyces hansenii. FEMS Yeast Res.

[21] Reifenberger E, Boles E, Ciriacy M (1997). Kinetic characterization of individual hexose transporters of *Saccharomyces cerevisiae* and their relation to the triggering mechanisms of glucose repression. Eur J Biochem.

[22] Diderich JA, Schepper M, van Hoek P, Luttik MAH, van Dijken JP, Pronk JT (1999). Glucose uptake kinetics and transcription of HXTGenes in chemostat cultures of *Saccharomyces cerevisiae*. J Biol Chem.

[23] Young E, Poucher A, Comer A, Bailey A, Alper H (2011). Functional survey for heterologous sugar transport proteins, using *Saccharomyces cerevisiae* as a host. App Environ Microbiol.

[24] Maier A, Völker B, Boles E, Fuhrmann GF (2002). Characterisation of glucose transport in *Saccharomyces cerevisiae* with plasma membrane vesicles (countertransport) and intact cells (initial uptake) with single Hxt1 Hxt2 Hxt3 Hxt4 Hxt6 Hxt7 or Gal2 transporters. FEMS Yeast Res.

[25] Sun L, Zeng X, Yan C, Sun X, Gong X, Rao Y (2012). Crystal structure of a bacterial homologue of glucose transporters GLUT1–4. Nature.

[26] Wambo TO, Chen LY, Phelix C, Perry G (2017). Affinity and path of binding xylopyranose unto E. coli xylose permease. Biochem Biophys Res Commun.

[27] Madej MG, Sun L, Yan N, Kaback HR (2014). Functional architecture of MFS D-glucose transporters. Proc Natl Acad Sci.

[28] Shin HY, Nijland JG, de Waal PP, de Jong RM, Klaassen P, Driessen AJM (2015). An engineered cryptic Hxt11 sugar transporter facilitates glucose-xylose co-consumption in *Saccharomyces cerevisiae*. Biotechnol Biofuels.

[29] Subtil T, Boles E (2012). Competition between pentoses and glucose during uptake and catabolism in recombinant *Saccharomyces cerevisiae*. Biotechnol Biofuels.

[30] Donzella L, Varela JA, Sousa MJ, Morrissey JP (2021). Identification of novel pentose transporters in Kluyveromyces marxianus using a new screening platform. FEMS Yeast Res.

[31] Hector RE, Qureshi N, Hughes SR, Cotta MA (2008). Expression of a heterologous xylose transporter in a *Saccharomyces cerevisiae* strain engineered to utilize xylose improves aerobic xylose consumption. Appl Microbiol Biotechnol.

[32] de Sales BB, Scheid B, Gonçalves DL, Knychala MM, Matsushika A, Bon EPS (2015). Cloning novel sugar transporters from Scheffersomyces (Pichia) stipitis allowing d-xylose fermentation by recombinant *Saccharomyces cerevisiae*. Biotechnol L.

[33] dos Reis TF, de Lima PBA, Parachin NS, Mingossi FB, de Castro Oliveira JV, Ries LNA (2016). Identification and characterization of putative xylose and cellobiose transporters in Aspergillus nidulans. Biotechnol Biofuels BioMed Cent.

[34] Lane S, Xu H, Oh EJ, Kim H, Lesmana A, Jeong D (2018). Glucose repression can be alleviated by reducing glucose phosphorylation rate in *Saccharomyces cerevisiae*. Sci Rep.

[35] Caballero A, Ramos JL (2017). Enhancing ethanol yields through D-xylose and L-arabinose co-fermentation after construction of a novel high efficient L-arabinose-fermenting *Saccharomyces cerevisiae* strain. Microbiology.

[36] Li H, Schmitz O, Alper HS (2016). Enabling glucose/xylose co-transport in yeast through the directed evolution of a sugar transporter. App Microbiol Biotechnol.

[37] Wang M, Yu C, Zhao H (2016). Directed evolution of xylose specific transporters to facilitate glucose-xylose co-utilization. Biotechnol Bioeng.

[38] Nijland JG, Shin HY, de Jong RM, de Waal PP, Klaassen P, Driessen AJM (2014). Engineering of an endogenous hexose transporter into a specific D-xylose transporter facilitates glucose-xylose co-consumption in *Saccharomyces cerevisiae*. Biotechnol Biofuels.

[39] Kuanyshev N, Deewan A, Jagtap SS, Liu J, Selvam B, Chen LQ (2021). Identification and analysis of sugar transporters capable of co-transporting glucose and xylose simultaneously. Biotechnol J.

[40] Young EM, Comer AD, Huang H, Alper HS (2012). A molecular transporter engineering approach to improving xylose catabolism in *Saccharomyces cerevisiae*. Metabol Eng.

[41] Wijsman M, Marques WL, Hettinga JK, van den Broek M, de la CortésCortTorre P, Mans R (2019). A toolkit for rapid CRISPR-SpCas9 assisted construction of hexose-transport-deficient *Saccharomyces cerevisiae* strains. FEMS Yeast Res.

[42] Reider Apel A, Ouellet M, Szmidt-Middleton H, Keasling JD, Mukhopadhyay A (2016). Evolved hexose transporter enhances xylose uptake and glucose/xylose co-utilization in *Saccharomyces cerevisiae*. Sci Rep.

[43] Lin Z, Li WH (2011). Expansion of hexose transporter genes was associated with the evolution of aerobic fermentation in yeasts. Mol Biol Evol.

[44] Wohlbach DJ, Kuo A, Sato TK, Potts KM, Salamov AA, Labutti KM (2011). Comparative genomics of xylose-fermenting fungi for enhanced biofuel production. Proc Natl Acad Sci USA.

[45] Riley R, Haridas S, Wolfe KH, Lopes MR, Hittinger CT, Göker M (2016). Comparative genomics of biotechnologically important yeasts. Proc Natl Acad Sci.

[46] Borelli G, Fiamenghi MB, dos Santos LV, Carazzolle MF, Pereira GAG, José J (2019). Positive selection evidence in xylose-related genes suggests methylglyoxal reductase as a target for the improvement of yeasts’ fermentation in industry. Genome Biol Evolution.

[47] Bueno JGR, Borelli G, Corrêa TLR, Fiamenghi MB, José J, de Carvalho M (2020). Novel xylose transporter Cs4130 expands the sugar uptake repertoire in recombinant *Saccharomyces cerevisiae* strains at high xylose concentrations. Biotechnol Biofuels.

[48] Lin HH, Han LY, Cai CZ, Ji ZL, Chen YZ (2006). Prediction of transporter family from protein sequence by support vector machine approach. Proteins.

[49] Li H, Dai X, Zhao X (2008). A nearest neighbor approach for automated transporter prediction and categorization from protein sequences. Bioinformatics.

[50] Bhasin M, Raghava GPS (2004). Classification of nuclear receptors based on amino acid composition and dipeptide composition. J Biol Chem.

[51] Chou KC, Cai YD (2002). Using functional domain composition and support vector machines for prediction of protein subcellular location. J Biol Chem.

[52] Sarda D, Chua GH, Li K, bin, Krishnan A. (2005). pSLIP: SVM based protein subcellular localization prediction using multiple physicochemical properties. Bioinformatics.

[53] Lv Z, Jin S, Ding H, Zou Q (2019). A random forest sub-golgi protein classifier optimized via dipeptide and amino acid composition features frontiers in bioengineering and biotechnology. Frontiers.

[54] Gromiha MM, Yabuki Y (2008). Functional discrimination of membrane proteins using machine learning techniques. Bioinformatics.

[55] Emms DM, Kelly S (2015). OrthoFinder: solving fundamental biases in whole genome comparisons dramatically improves orthogroup inference accuracy. Genome Biol.

[56] Bateman A, Martin MJ, O’Donovan C, Magrane M, Alpi E, Antunes R (2017). UniProt: the universal protein knowledgebase. Nucleic Acids Res.

[57] Saier MH, Tran CV, Barabote RD (2006). TCDB: the transporter classification database for membrane transport protein analyses and information. Nucleic Acids Res.

[58] Pedregosa F, Varoquaux G, Gramfort A, Michel V, Thirion B, Grisel O (2012). Scikit-learn: machine learning in python. J Mach Learn Res.

[59] Gromiha MM (2010). Protein sequence analysis. protein. Bioinformatics.

[60] Dayhoff MO, Schwartz RM (1978). Chapter 22: a model of evolutionary change in proteins. Atlas of protein sequence and structure.

[61] Bigelow CC (1967). On the average hydrophobicity of proteins and the relation between it and protein structure. J Theor Biol.

[62] van Westen GJP, Swier RF, Cortes-Ciriano I, Wegner JK, Overington JP, Jzerman API (2013). Benchmarking of protein descriptor sets in proteochemometric modeling (part 2): modeling performance of 13 amino acid descriptor sets. J Cheminform.

[63] Chou KC (2005). Using amphiphilic pseudo amino acid composition to predict enzyme subfamily classes. Bioinformatics.

[64] Mistry J, Chuguransky S, Williams L, Qureshi M, Salazar GA, Sonnhammer ELL (2021). Pfam: The protein families database in 2001. Nucleic Acids Res.

[65] Bellasio M, Peymann A, Steiger MG, Valli M, Sipiczki M, Sauer M (2016). Complete genome sequence and transcriptome regulation of the pentose utilizing yeast Sugiyamaella lignohabitans. FEMS Yeast Res.

[66] Trichez D, Steindorff AS, Soares CEVF, Formighieri EF, Almeida JRM (2019). Physiological and comparative genomic analysis of new isolated yeasts *Spathaspora* sp JA1 and Meyerozyma caribbica JA9 reveal insights into xylitol production. FEMS Yeast Res.

[67] Mixao V, Hegedusova E, Saus E, Pryszcz LP, Cillingova A, Nosek J (2021). Genome analysis of Candida subhashii reveals its hybrid nature and dual mitochondrial genome conformations. DNA Res.

[68] Runquist D, Fonseca C, Rådström P, Spencer-Martins I, Hahn-Hägerdal B (2009). Expression of the Gxf1 transporter from Candida intermedia improves fermentation performance in recombinant xylose-utilizing *Saccharomyces cerevisiae*. Appl Microbiol Biotechnol.

[69] Baek M, DiMaio F, Anishchenko I, Dauparas J, Ovchinnikov S, Lee GR (1979). Accurate prediction of protein structures and interactions using a three-track neural network. Science.

[70] Ramírez D, Caballero J (2018). Is It reliable to take the molecular docking top scoring position as the best solution without considering available structural data?. Molecules.

[71] Mishra NK, Chang J, Zhao PX (2014). Prediction of membrane transport proteins and their substrate specificities using primary sequence information. PLoS ONE.

[72] Haas BJ, Salzberg SL, Zhu W, Pertea M, Allen JE, Orvis J (2008). Automated eukaryotic gene structure annotation using evidencemodeler and the program to assemble spliced alignments. Genome Biol.

[73] Drew D, North RA, Nagarathinam K, Tanabe M (2021). Structures and general transport mechanisms by the major facilitator superfamily (MFS). Chem Rev.

[74] Wisedchaisri G, Park M-S, Iadanza MG, Zheng H, Gonen T (2014). Proton-coupled sugar transport in the prototypical major facilitator superfamily protein XylE. Nat Commun.

[75] Blomqvist J, South E, Tiukova L, Momeni MH, Hansson H, Ståhlberg J (2011). Fermentation of lignocellulosic hydrolysate by the alternative industrial ethanol yeast dekkera bruxellensis. Lett Appl Microbiol.

[76] Senatham S, Chamduang T, Kaewchingduang Y, Thammasittirong A, Srisodsuk M, Elliston A (2016). Enhanced xylose fermentation and hydrolysate inhibitor tolerance of scheffersomyces shehatae for efficient ethanol production from non-detoxified lignocellulosic hydrolysate. Springerplus.

[77] Carvalho LM, Carvalho-Netto OV, Calderón LL, Gutierrez M, de Assis MA, Mofatto LS (2021). Understanding the differences in 2G ethanol fermentative scales through omics data integration. FEMS Yeast Res.

[78] Keller O, Kollmar M, Stanke M, Waack S (2011). A novel hybrid gene prediction method employing protein multiple sequence alignments. Bioinformatics.

[79] Besemer J, Lomsadze A, Borodovsky M (2001). GeneMarkS a self-training method for prediction of gene starts in microbial genomes implications for finding sequence motifs in regulatory regions. Nucl Acids Res.

[80] Simão FA, Waterhouse RM, Ioannidis P, Kriventseva EV, Zdobnov EM (2015). BUSCO: assessing genome assembly and annotation completeness with single-copy orthologs. Bioinformatics.

[81] Katoh K, Standley DM (2013). MAFFT multiple sequence alignment software version 7: improvements in performance and usability. Mol Biol Evol.

[82] Ranwez V, Harispe S, Delsuc F, Douzery EJP (2011). MACSE: multiple alignment of coding sequences accounting for frameshifts and stop codons Murphy WJ, editor. PLoS ONE.

[83] Capella-Gutiérrez S, Silla-Martínez JM, Gabaldón T (2009). TrimAl: a tool for automated alignment trimming in large-scale phylogenetic analyses. Bioinformatics.

[84] Minh BQ, Schmidt HA, Chernomor O, Schrempf D, Woodhams MD, von Haeseler A (2020). IQ-TREE 2: new models and efficient methods for phylogenetic inference in the genomic Era. Mol Biol Evol.

[85] Murrell B, Wertheim JO, Moola S, Weighill T, Scheffler K (2012). Detecting individual sites subject to episodic diversifying selection. PLoS Genet.

[86] Li W, Godzik A (2006). Cd-hit: a fast program for clustering and comparing large sets of protein or nucleotide sequences. Bioinform Oxf Acad.

[87] Xiao N, Cao DS, Zhu MF, Xu QS (2015). Protr/ProtrWeb: R package and web server for generating various numerical representation schemes of protein sequences. Bioinformatics.

[88] Sigrist CJA, de Castro E, Cerutti L, Cuche BA, Hulo N, Bridge A (2013). New and continuing developments at PROSITE. Nucl Acids Res.

[89] de Castro E, Sigrist CJA, Gattiker A, Bulliard V, Langendijk-Genevaux PS, Gasteiger E (2006). ScanProsite: detection of PROSITE signature matches and ProRule-associated functional and structural residues in proteins. Nucl Acids Res.

[90] Chen T, Guestrin C. 2016 XGBoost: a scalable tree boosting system. 10.1145/2939672.2939785.

[91] Bengfort B, Bilbro R, Danielsen N, Gray L, McIntyre K, Roman P, et al. 2018 Yellowbrick v0.9 https://zenodo.org/record/1488364.

[92] Lundberg SM, Lee SI (2017). A unified approach to interpreting model predictions. Adv Neural Inf Process Syst.

[93] Mcinnes L, Healy J, Melville J (2018). UMAP: Uniform manifold approximation and projection for dimension reduction. J Open Sour Softw.

[94] Gietz RD (2014). Yeast Transformation by the LiAc/SS Carrier DNA/PEG Method.

[95] Bell EW, Zhang Y (2019). DockRMSD: an open-source tool for atom mapping and RMSD calculation of symmetric molecules through graph isomorphism. J Cheminform.

[96] Schöning-Stierand K, Diedrich K, Fährrolfes R, Flachsenberg F, Meyder A, Nittinger E (2020). ProteinsPlus: interactive analysis of protein–ligand binding interfaces. Nucleic Acids Res.

