# Supplementary material for: Machine learning and comparative genomics approaches for the discovery of xylose transporters in yeast
Source: Biotechnol Biofuels Bioprod. 2022 May 20;15:57. doi: 10.1186/s13068-022-02153-7 (PMC9123741; doi:10.1186/s13068-022-02153-7)
Supplement: Supplementary file 6 — Additional file 6: Figure S1. BUSCO results for gene prediction. [file 13068_2022_2153_MOESM6_ESM.docx]

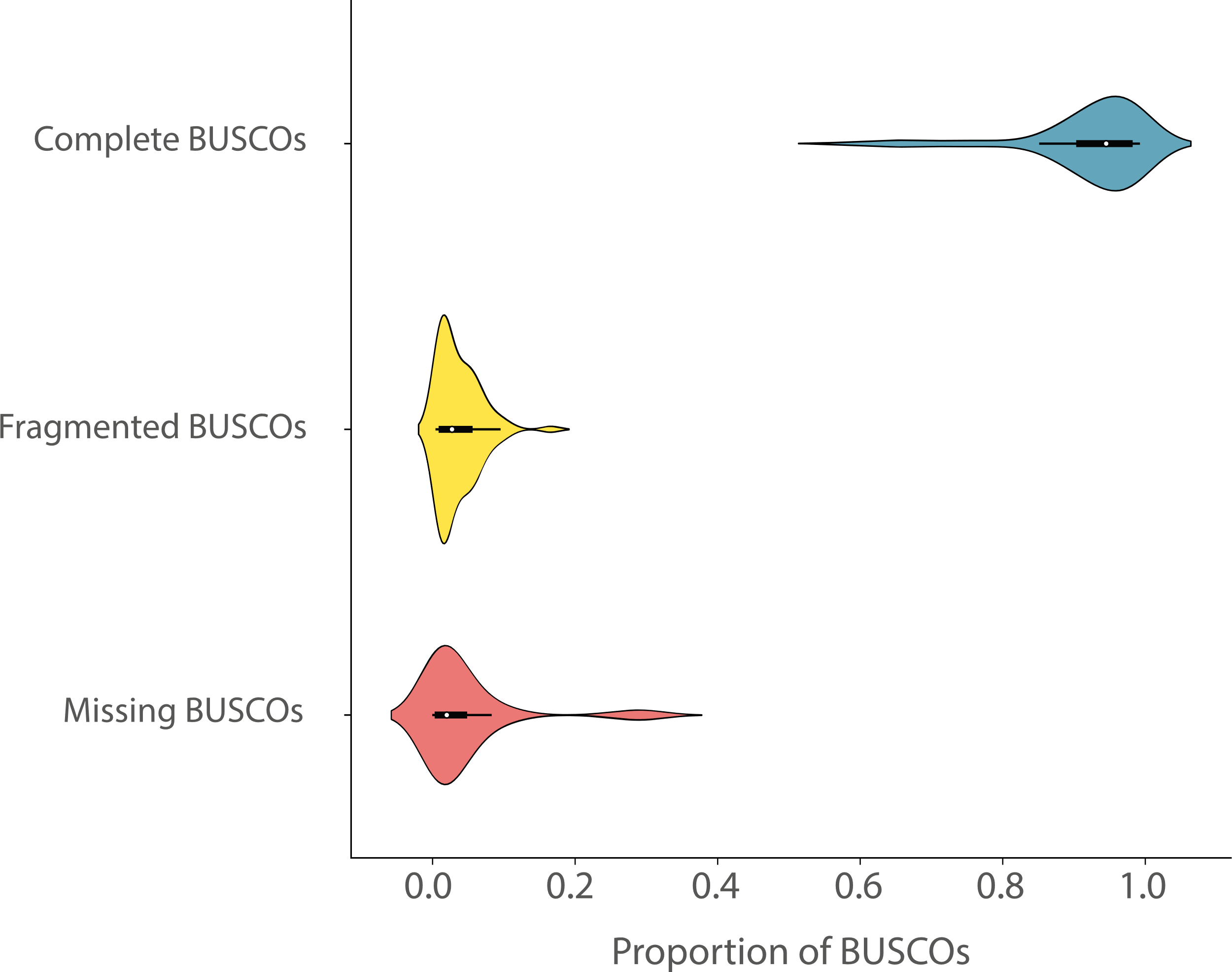


**Supplementary Figure 1.** Violin-plot of BUSCO results for gene prediction. The graph highlights that most BUSCOs were found complete (close to 90%), while a low percentage (less than 30%) were found fragmented or were missing
