# Supplementary material for: Machine learning and comparative genomics approaches for the discovery of xylose transporters in yeast
Source: Biotechnol Biofuels Bioprod. 2022 May 20;15:57. doi: 10.1186/s13068-022-02153-7 (PMC9123741; doi:10.1186/s13068-022-02153-7)
Supplement: Supplementary file 7 — Additional file 7: Figure S2. UMAP spatial distribution of data after oversampling. [file 13068_2022_2153_MOESM7_ESM.docx]

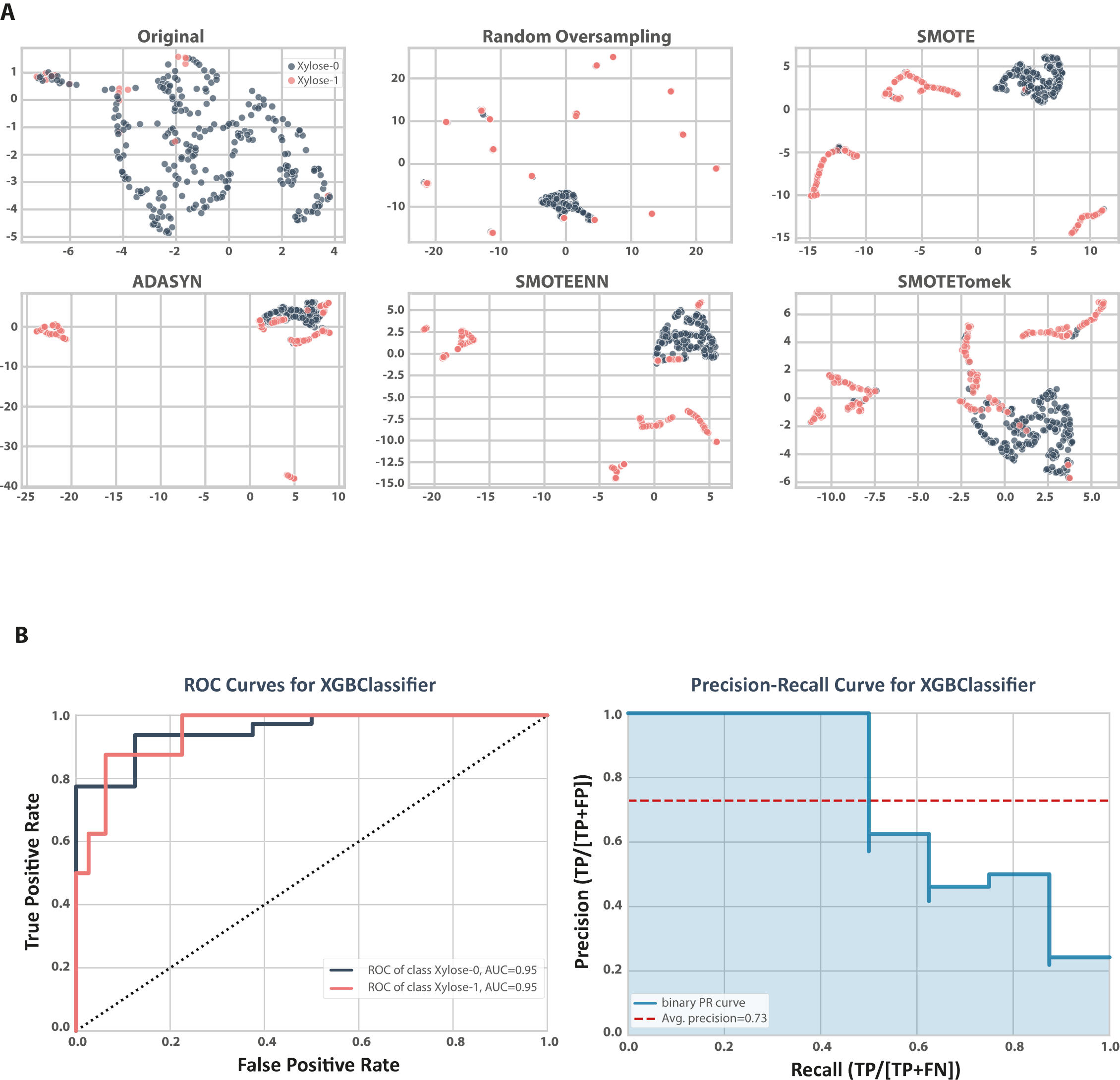


**Supplementary Figure 2**. a) UMAP spatial distribution of data after oversampling, through different methods; b) ROC and Precision-Recall curves of XGBoost after resampling with SMOTEENN.
